# On the Epidemic Cholera of Newburn

**Published:** 1832-07-01

**Authors:** 


### On the Epidemic Cholera of Newburn. By *D. Craigie*, M.D. (Ed. Journ. April 1832.)

From the accounts which appeared in the Scotch newspapers, and the proceedings of the Board of Health in Modern Athens, we had begun to despair of any gleam of light or reason from the city on the hills. The medical faculty on the north side of the Tweed seemed to embrace, one and all, the darkest and most gloomy doctrines of contagion that were ever propagated, at the point of the bayonet, on the banks of the Wolga, or amid the steep defiles of the Carpathian Mountains! In despite of this, and of the universal cry of contagion in this country, from the Privy Council in Whitehall, to the rudest cholera hospital in Musselburgh, we maintained the doctrine with which we set out—that cholera is an epidemic disease, and its propagation by contagion is comparatively a rare contingency, and unessential to its progress in this or in any other country. We regretted to find that so able and influential a periodical as the Edinburgh Medical and Surgical Journal had, with its usual candour and impartiality—and after weighing well the evidence transmitted to us from the Continent, come to the conclusion that cholera was a contagious disease, which had travelled from the Ganges to the Wear by means of personal communication—then, indeed, we perceived that we stood nearly alone, and that it would be almost a miracle if the tide of professional and popular belief did not run uninterruptedly against us. In Edinburgh, too, where we expected the most rigid scrutiny into facts, and the most severe and pure deductions from those facts, we learnt, to our surprise, that Mussulmen did not place more implicit faith in the Khoran, than did the magnates of the intellectual city in contagion, and contagion only! But we were mistaken. Those men who thought least, were loudest in their adhesion to the doctrines of the London boards of health—while others, who were investigating facts, and deeply meditating on the subject of the epidemic disease, were preparing to come forth with arguments and histories, calculated to disturb a little the premature professions of faith promulgated by their less cautious brethren. The paper which lies before us will make a strong impression on the minds of medical men; not only on account of the talents of its author, but of the spirit of philosophy and candour which pervades every line of the document. No one will say that Dr. Craigie came to this practical inquiry with a mind bi-assed in favour of non-contagion. If there was any leaning, it was to the other side, grounded on the faith of what he had *read*. We shall soon see how far the evidence of his own senses corroborated the second-hand evidence derived from others.

In no part, perhaps, of the world has the epidemic, called cholera, shewed more destructive ravages than in the village of Newburn, situated on the north bank of the Tyne, five or six miles from Newcastle. Thither Dr. Craigie repaired to view the disease with his own eyes, and to draw deductions from the evidence of his own senses. It appears that the north bank of the Tyne, at the place above-mentioned, rises abruptly into hills of moderate elevation, on the slope of which, between the river and these eminences, stands the village of Newburn. The opposite or south side of the river is a flat alluvial soil. The following passage will convey some idea of the medical topography of the north bank of the Tyne, where are situated Scottswood, Limmington, and Newburn.

"Many, if not most of them (the houses) are built on a descending bank, in such a manner that, though the southern exposure of the house is above ground, from four to five feet at least, the northern or back exposure is below the surface of the earth. The doors of these houses, which are on their northern sides, are at the base of a flight of five or six steps, and the windows, which are not about three feet high, are completely on a level with the surface of the ground. A pathway about five feet broad runs along them; and on the north side of the path is a row of small hovels constructed of wood and clay or earth, and devoted chiefly to the breeding of pigs and some poultry. In this tract humidity abounds, and there is a good deal more filth than is compatible either with comfort or salubrity." 339.

Nevertheless the inhabitants of these villages appear to have enjoyed good health, the staple food being bacon, and bread baked with milk and butter. The village of Newburn contains 131 houses, 13 of which contained two families each. The number of inhabitants was estimated at 550. In the latter end of December, a young man was attacked with the epidemic, went through the diarrhœal and asphyxial stages without remedies, and died, on the 4th January of the present year, of the consecutive fever, apparently a cerebral inflammation. Popular rumour connected this case, as usual, with importation from Newcastle, in the person of a man named Wailes, who had gone to Newcastle and returned to *Limmington* some time about the death of his son in Newcastle. The widow of the son had also come to Limmington, where the father in-law, an intemperate glass-blower, exposed to great vicissitudes of climate, was attacked, and died on the 29th December, the very day when Robson was attacked at Newburn. It is needless to dwell on such ridiculous proofs of the introduction of the contagion as these. It is of far more importance to state, that "diarrhœa had been prevailing to a greater or less extent, among all the houses situated along the north bank of the Tyne, from the middle till the end of December, at a period when it was impossible to suppose that it was transferred from the person of Wailes, and when it was known that several of the associates of Robson were labouring under choleric or common diarrhœa." The following passage offers a remarkable corroboration of the statement which we published in our Journal for January, page 304.

"I then learnt, in a very partial and imperfect manner, a fact of which I have since had at Musselburgh tolerably satisfactory proof, that in many persons slight looseness comes on, and after lasting for a day or two subsides spontaneously, or after very ordinary domestic remedies, and, if not re-excited by some error in diet or exposure to cold or moisture, permanently. But after any irregularity, however slight, this looseness is liable to recur, and again subside, or under certain favourable circumstances to be converted into cholera, mild or virulent. *I must also say in reference to this subject, that, however proper, symptomatically and therapeutically, the distinction of cases into* diarrhœa, common cholera, *and* malignant cholera *may appear, it is, when applied to the epidemic, altogether inadmissible. I am satisfied, from the progress of this epidemic both on the Continent, and in England and Scotland, and from the facts which I have been able to collect either personally or from others, that these diseases differ in degree only, that all of them depend on common causes operating in different degrees, in different localities, and on different individuals.*" 343.

Dr. Craigie afterwards adds :—"We must, therefore, I conceive, admit a tendency to gastro-enteric disorder, which assumes, in certain classes of persons, different forms, *until it attains the specific degree of virulence* constituting genuine cholera." We request our readers to turn to the page above indicated, and see the remarkable coincidence of opinion between Dr. Craigie and ourselves.

We do not deem it necessary to follow our highly gifted author through the minute history of the epidemic as it spread over, or rather prevailed in, the devoted village of Newburn. We shall, however, introduce the celebrated proof of contagion afforded by the death of the Rev. Mr. Edmonston, Vicar of Newburn.

"This day was, however, remarkable for the 15th case, and the 5th death, which deserves particular note, being that of the vicar of the parish, the Rev. Mr. Edmonston, which has been adduced by several persons as an instance of the influence of contagion. This idea I think it quite impossible to adopt, if we consider the circumstances of the case with any attention; but to obviate any thing like bias, I shall state the simple facts, as I know well they took place.

The Reverend Mr. John Edmonston was a man somewhere between 76 and 80, of a full habit, and rather inclined to corpulence. He had, indeed, been somewhat among the sick; but this must have been of short duration, and was only during the last two or three days, that is, not sooner than Thursday the 12th of the month; and as he was extremely afraid of the disease, there was little reason to believe that he would expose himself unnecessarily. If I am not misinformed, indeed, he took every precaution in approaching the sick which a person impressed with the firm belief of the contagious nature of the disease could employ. His house, I must further remark, is situated at the west extremity of the Tyne side of the village, much nearer the river than any of the others, and stands, indeed, on a declining bank, with nothing but the terrace of the railway interposed between it and the water. Though extremely clean inside, snug and comfortable, I must say the situation is such as to obstruct in a remarkable degree the process of free perflation and ventilation. With the artificial terrace of the railway on the south, and the rising bank of the village and churchyard on the north, the situation of the vicarage of Newburn is such as to favour in a remarkable manner the sojourn of any damp vapours which might pass from the river or its banks to the adjoining surface. To this may be added, the circumstance, that the whole is surrounded by a high wall, which, on the one hand, ought to have excluded contagious principles or their conductors, while, on the other, it prevented the free circulation of atmospherical currents.

Mr. Edmonston was a scientific and amateur horticulturist, and was particularly successful in rearing some of the more rare and delicate varieties of tree and wall fruit; part of which he was in the habit of presenting to friends, part of using at his own table. For three weeks previous he had abstained from the latter indulgence with the rigour of an ascetic, and the self-denial of a philosopher. On Saturday the 14th of January, however, his circumspection appears to have been thrown off its guard; for on that day he had dined on an inauspicious dish of pickled salmon,—an article which is sufficient of itself in some constitutions to induce an attack of cholera, and which, in the present case, aided by the epidemic constitution, was too successful in giving rise to a rapid and violent attack of the epidemic disease. Mr. Edmonston appears to have felt no uneasiness for some hours after dinner. But he retired to his chamber at the usual time; and his domestics, who heard nothing of him till morning, found him then in a state of great debility and helplessness. He had been attacked with sickness soon after going to bed, and the whole night he had been purged repeatedly and violently. The reverend gentleman had some knowledge of medicine himself; and whether it was from distrust of its powers, or from a desperate idea of his own case, or from approaching insensibility, for he was of what is denominated an apoplectic habit, he neither asked for assistance, nor, when his servant proposed it, would he hear of it. He had also, it must be acknowledged, some unreasonable prejudice against our excellent friend Mr. Fyfe, whose delicacy would not allow him to wound the old gentleman's feelings by his presence. After some deliberation, however, it was determined that some attempt should be made to arrest the progress of his disorder; and accordingly he was visited first by Dr. Cobb, who prescribed the usual remedies, and then by Mr. Fyfe and Dr. Cobb together. At the first visit, about half-past two, his face was flushed, his sensibility impaired, and his pulse slow and labouring, and he seemed to be suffering under symptoms of cholera complicated with those of oppressed brain. He had occasional retching, and frequent rice-water dejections. An hour after, the symptoms of vascular excitement began to give way to those of colliquation. The face was bluish and leaden coloured; cold clammy perspiration poured from every part of the surface; he had frequent hiccup; the sero-albuminous dejections continued to run from him; and the spasms of the legs were repeated and violent. Mr. Fyfe and Dr. Cobb remained with him to the last, using every possible means to avert the fatal termination; but the old man breathed his last a little before five o'clock, not more than twenty hours from the first symptoms of uneasiness, and about twenty-five or twenty-six hours after the repast which had manifestly operated as the exciting cause.

I have been thus particular in giving the details of this case, because it has been more than once appealed to as an example of communication of the disease from the sick to the sound. I feel no difficulty in stating my opinion, founded on the reasons already assigned, that it is sufficient to look to the plethoric habits of the individual, the imprudent error of diet, and the epidemic constitution hanging over the village, and from which it was impossible that the parsonage could be exempt, in order to account for the attack and its unhappy termination. Where the attack can be explained on manifest principles, it is entirely gratuitous to have recourse to such an occult cause as contagion. *I venture to assert, without possibility of refutation, that the intercourse with the sick went in this case for nothing; and, had Mr. Edmonston secluded himself within his garden wall, the pickled salmon would have produced precisely the same effect.*" 349.

On the 16th of January, there were at least *fifty* new cases, in a population of 550! This is indeed a remarkable instance of a disease communicated from person to person! The following passage deserves to be recorded in brass or marble—but perhaps it will be more durable in ink.

"I went to Newcastle, however, with the determination to throw aside every thing I had learnt from the writings of others; to divest my mind of every preconceived notion; to doubt every thing regarding it; and to observe the disease personally with the impression that every fact required to be ascertained. I am quite aware that by this avowal I expose myself to the imputation of some degree of arrogance in supposing that all previous observers had been deficient in accuracy or assiduity, or had somehow failed to communicate a correct delineation of the malady. I have only to reply, that I went to study the disease personally; and as I confess my mind was a good deal confused by the discordant accounts of the success of very opposite modes of treatment, I was desirous to endeavour, on my own account, to disentangle some of the perplexity, and to form something like a distinct idea of the best and most efficient mode of treatment, in the event of the disease arriving, as it has since done, in our neighbourhood. Even on the great question, of the mode in which the disease originates and is propagated, I studied to go like one unbiassed either to the one side or the other, and resolved to admit no inference, unless supported by unambiguous facts." 356.

In the first place Dr. Craigie doubts the general opinion that the disease attacks *only* "the feeble, the broken down, and the dissipated, or even those in indigent circumstances." We agree entirely with Dr. C. on this point; but we maintain that, generally speaking, it is in the above classes, we have the exquisite and fatal forms of the malady. Among the affluent, as we stated in our last number, the peer may occasionally place himself on a level with the peasant, by predisposition, or by locality. This does not affect the general question as to predisposition. Dr. Craigie attaches great importance to errors of diet, during the prevalence of an epidemic affecting the digestive organs ; and justly so. In the great majority of instances which we have investigated in this metropolis, we have traced the *exciting* cause to this source, both in the poor and the rich. He who is blind to this exciting cause is not an accurate observer.

" In a very large proportion of cases the disease began with a sense of weight and fulness in the epigastric region, suddenly followed by profuse diarrhœa. The rapidity with which this symptom proceeded to the true choleric discharges varied in different cases, and appeared to depend on the constitutional powers of the individual, on the situation in which he was living, and on the promptitude with which the diarrhœa was mitigated and controlled by appropriate remedies. Thus in the case of the Reverend Mr. Edmonston, in whom the diarrhœal stage was extremely short, the individual was aged, plethoric, and neglected all remedies of every kind, so that till 2 o'clock, p. m. on the 15th, it may be regarded as an example of the uncontrolled and unmodified progress of the disease. In the case of Mary Newton, on the contrary, who was young, vigorous, and of unbroken constitution, but labouring under deep and overpowering mental distress, on account of the hopeless state of her sister, and the numerous instances of mortal sickness around her, the diarrhœal stage lasted from Sunday afternoon about 3, till the following day about 2, very nearly 24 hours. In the case of Mary Gibson, again, in whom the disease never assumed the genuine choleric symptoms, after anomalous illness of a week's duration, purging took place on Wednesday 11th, the second day of the epidemic, then some vomiting, after which she recovered under the use of some medicine ; then on Saturday the 14th, she had discharges of watery fluid from the bowels ; and on Monday, when we saw her, she complained chiefly of weakness and faintness, but was then convalescent.

In other cases, however, the symptoms observed a different course, and after preserving the diarrhœal form for several days, subsided so much as to lead to the belief that the patient was convalescent, suddenly assumed the choleric character, with profuse and repeated rice-water discharges, sinking of the pulse, and coldness of the surface, and terminated fatally in the course of 20 or 24 hours." 359.

Dr. C. observes that the transition from the diarrhœal stage to that of collapse, was never, as it were *per saltum ;* though sometimes rapid. In all cases, there were observed " gradual and successive changes."

" In most of the cases in which I had an opportunity of remarking this transition, the countenance became first slightly blanched, and the skin began to assume a colliquative humidity. When the pulse was felt at this period, it was not gone, but greatly weakened in force and small in its size. The patient at the same time complained of a sense of sinking at the heart, with an uncomfortable sense of thrilling heat and unsteadiness, as if unable to support himself ; and though there were instances in which the patient fell down at this period from weakness, yet afterwards, when the stage of collapse was thoroughly established, this extreme enfeeblement of the voluntary muscles was not recognized.

When the stage of collapse is fully established, the countenance is not so much of a blue colour as of a dirty, or dingy leaden tint. The only part which makes any approach to blueness is the *prolabia,* or red skin of the lips, which in several cases I certainly allow did assume the tint of the bilberry,—and the nails, which are livid, or of a purple blue. At the same time, the skin of the arms and hands, and legs and feet became corrugated, or puckered into wrinkles ; and the hands and fingers became rigidly and immoveably incurvated, as if the flexor muscles were affected by tetanic spasms. This symptom I regard as by far the most characteristic of the stage of collapse ; for I remember none in which it was wanting; and I saw several in which the others were not marked till immediately before death." 361.

Vomiting generally ceases when the stage of collapse is fully formed; but the discharges from the bowels usually continue to run off till the last. Dr. C. tested this fluid in many cases, but in one only did it produce any change, turning turmeric paper brown, and thereby indicating alkalescent qualities. Professor Turner found it to contain much albumen, and the salts of the serum.

The most prominent phenomenon in this disease, says Dr. C. is the failure of the circulation. Yet Dr. C. found the action of the heart to be always violent, jarring, and tumultuous, even when there was no pulse at the wrist. This we have frequently observed, by means of the stethoscope, after it had been "put down" in notes, at the bed-side, that the heart's action had ceased! It is not a little strange, that even the Central Board of Health has fallen into this error, and talk of the cessation of the heart's action long before death.

"Suppression of the urinary secretion, though frequent, is not an invariable symptom. There is no doubt that it is extremely scanty, and perhaps before death it may be altogether suppressed. But both at Newburn and Newcastle we met with instances in which the urine continued to be secreted during part at least of the stage of collapse. In the case of Margaret Jameson, a woman of 44, which was particularly well-marked, and terminated fatally, both the patient and her sister asserted, that she continued to void urine 12 hours at least after the commencement of the state of collapse, 2 days before death, while the surface was leaden-coloured, sodden, and cold, cramps were violent, and retching and purging were frequent and profuse." 363.

We do not know that a circumstance which we have more than once observed, namely, a discharge from the bowels of a fluid exactly resembling urine, both in colour and smell, has been noticed by any writers.

The majority of the cases were cut off in the stage of collapse—several in the consecutive fever. Dr. Craigie considers this fever as very different from typhus. In the anastasis, or consecutive fever of cholera, the heat is rarely above par; and though the action of the heart is strong, that of the arteries is feeble. The vessels of the head are invariably congested—and, in this respect, the term *typhoid* may be fairly applied, for who sees typhus without similar affection? The consecutive fever was always in proportion to the length and violence of the preceding collapse or cold stage, just as we see in intermittents of bad kind.

Out of 550 inhabitants, 320 had suffered from the epidemic; and of these 55 died, or one-tenth of the whole population!

On the pathology of this mysterious disease, Dr. Craigie has hazarded some ingenious speculations. We shall allow him to speak for himself.

"On the obscure and perplexing subject of the pathology of cholera I have little to say. The lesion of the nervous system, which has been so generally assumed, I confess, appeared to me nowhere to exist. It was clear that neither the brain, nor the spinal chord, or their nerves were *peculiarly* affected; and I could see as little proof that the gangliar system or the solar plexus was pre-eminently disordered. These organs, indeed, partake of the general lesion of the blood-vessels, in common with all others to which blood-vessels are distributed; but that they are specifically affected is, I conceive, a hypothetical assumption entirely.

All the phenomena of cholera, on the contrary, indicate in my opinion an affection of the vascular system and its contents; and in the exquisite form of the disease it bears a close resemblance to inanition of these vessels, *anaemia* suddenly induced, or the effects of profuse hemorrhagy. I do not mean to enter into any discussion of the question, whether the sero-albuminous discharge is a secretion, or by what mechanism it is effused. It cannot be doubted that it is poured forth from the blood-vessels of the intestinal tube, or the branches of the cœliac and mesenteric arteries at the expense of the serum, and much of the albumen of the blood. The effects of this are at least twofold. In the *first* place, the quantity of the circulating fluid is absolutely and most suddenly diminished; and *secondly*, by deprivation of its sero-albuminous portion, its fluidity is remarkably impaired, and it is thereby rendered less capable of moving, especially in the vessels of the third and fourth classes. These two circumstances combined give cholera a character consisting partly of hemorrhage or *syncope*, and partly *asphyxia;* and on this singular combination seems to depend the phenomenon of the cramps of the extremities, and the tonic incurvation of the hands, and fingers, and toes. Any one who has witnessed death by sudden hemorrhage, or has seen an animal bled to death, knows that spasmodic affections of the voluntary muscles accompany the last agonies of life. Those of genuine and exquisite cholera, I conceive, are of the same nature, and depend on a similar cause. As the blood is withdrawn from the muscles of the extremities to supply the defect in the large vessels communicating with the cœliac and mesenteric arteries, and as it ceases to move through them, these muscles are affected by irregular and involuntary contractions. On this subject, however, I cannot at present dwell longer." 372.

### Propagation of Cholera.

The foregoing quotations and abbreviations must shew our readers that Dr. Craigie *observed* for himself and observed very accurately too. His observations on the mode in which the disease is evolved, must convince them that he *thought* for himself also—and that in a very original and independent manner.

"The question of the mode in which cholera arises and is propagated is perhaps not less difficult and perplexing. *The summary method of resolving the whole into importation and contagion has been very generally adopted; and the successive progress of the disease from the Gangetic Delta to the British shores, in the course of* 14 *years, has been confidently adduced as the strongest proof of the soundness of this view.* Many rather hard names have been applied to those who have avowed their inability to admit the conclusiveness of this doctrine; and some even have expressed their astonishment at the construction of the minds of those who have ventured to doubt the justice of the hypothesis of importation and contagion. *I am not ashamed to say, that to this school I do not belong, and that to this logic I shall not have recourse.* Every question regarding the mode in which a disease originates, and is propagated among a community of human beings, *must* be matter of opinion and hypothesis, and cannot be decided either by the numbers or the weight of the names by which it is supported. It is only by a cautious comparison of well-authenticated facts, so unequivocal and unambiguous as to admit of one explanation only, that this point can be determined. I shall in the following observations state the facts with which my visit to Newcastle made me acquainted, and compare them shortly with those observed by others." 372.

We need not follow Dr. Craigie through the historical details, and the specific proofs, of non-importation into Newburn—the wide circulation of the Journal which he so ably conducts, will ensure his observations an ample range of readers.

The following passage will speak volumes.

"The disease appears in several persons insulated and unconnected with each other, either all at once in the same night, or within an incredibly short space of time, so that the idea of what I denominate efficient propagation is entirely out of the question. I presume that the 55 attacks in Gateshead, mostly in one street, and one side of it, on the night of the 25th and morning of the 26th, must be given up at once as utterly inexplicable on the principle of contagion; for I remember no instance in the whole course of the annals of disease, in which such a number of attacks is simultaneously produced by such an agent. I am, on the contrary, acquainted with many examples of numerous simultaneous attacks of disease depending on miasmatic or atmospherical causes, and presenting in this respect a close analogy to that of the invasion of the Gateshead epidemic. I shall simply advert to the remarkable one recorded by Lancisi, of 30 ladies and gentlemen of the first rank in Rome, who, on an excursion of pleasure to the mouth of the Tiber, were exposed to a sudden change of wind blowing from the south over the marshes, and 29 of them were immediately seized with tertian fever. Instances of this kind, however, which could be multiplied with great facility, have not for a long time, and are not now considered as proofs of contagion; and, indeed, it is given by Lancisi as an example of a morbific cause of a different kind entirely, that of *miasma*, or what has recently been termed *malaria;* and I presume that the Gateshead cause must either be at once relinquished, or ascribed to some similar agent.

The example of Newburn is equally strong, so far as a small place and a limited population can be compared with a large and populous one. Between the night of the 10th and morning of the 11th, 16 persons were attacked with diarrhœa or choleric symptoms, 13 of whom had pure cholera, and of these 12 died in the course of 30 hours. Nine of the pure choleric cases, it must also be remarked, were on the Tyne side of the village. The attacks went on at the rate of from 10 to 12 every night, with various degrees of severity: and before a revolution of seven days was completed, 36 persons had been cut off by the disease, or at the average rate of six daily. I must confess my ignorance of any contagious disease that appeared in this sudden manner, and made such rapid havoc among a community so limited; and I think no resource is left but to admit the spontaneous, the material, or the atmospherical origin of the malady.

With these facts, let us compare those attending the appearance of the disease in Astrachan, Moscow, and St. Petersburgh. Had cholera been capable of introduction, it is reasonable to think that it might be excluded by the rules of the seclusive and non-intercourse system. Each of these cities, accordingly, was placed under a rigid system of seclusion and separation from infected places, by means of strong lines of military posts and barriers, which in the case of Moscow were twofold. Notwithstanding these precautions, however, the disease appeared in every one of these cities, and without the possibility of tracing it in any instance to unequivocal importation.

The example of the kingdom of Hungary is perhaps still more forcible. Nor, perhaps, was such a rigid and perfect system of non-intercourse enforced as that which was instituted on the southern, northern, and eastern boundaries of Hungary, when the disease appeared in Poland. Every defile of the Carpathian mountains was guarded with strong military posts, and watched with the utmost vigilance. Neither human beings, goods, nor brute-beasts, except, perhaps, an occasional wolf or bear, could penetrate into the Hungarian territories, unless at the muzzle of the firelock or the point of the lance. Yet how vain these precautions proved, was evinced in the course of a few weeks. For the disease appeared on the Hungarian side of the Carpathian chain in the month of June, and spread most rapidly through the town situate on the banks of the rivers of that well-watered country. It will not do in this case to say that the sanitary lines were broken in the ordinary sense. Broken they no doubt were; but by no human power." 376.

Let the advocates of "travelling cholera"—of the disease being communicated from one individual to another, from the Ganges to the Wolga, from the Vistula to the Wear, read the above sentiments of Dr. Craigie.

Our able author examined, with a critical eye, the medical topography of Gateshead, Newburn, &c.; and offers many conclusive arguments, "that certain atmospheric conditions, operating on *peculiar localities*, are most likely connected with the origin of malignant cholera." How exactly these ideas coincide with our own, as stated in our last number, p. 675, the reader will easily judge.*

Dr. C. conceives that "no satisfactory proof has yet been adduced to show that the disease was imported from places where it was previously prevailing," and hence he regards it as unphilosophical "to assume a principle without sufficient evidence." The able author then goes on to investigate certain local peculiarities of situation where the disease has been most prevalent, in order that others may compare his observations with their own. For these we must refer to the original paper.

Reverting to the important subject of the propagation of cholera, Dr. C. remarks :—

"Though during the whole time of the epidemic, the intercourse between Newcastle and the neighbouring towns was extremely frequent and uninterrupted, no case of cholera had appeared. One example or two must suffice. There are few towns, perhaps, with which Newcastle has more intercourse than with Morpeth, an intercourse, I may add, conducted by persons who might act the part of very good and perfect conductors of any contagious disease. Regularly as the Wednesday returns, crowds of butchers and persons connected with the sheep and cattle trade flock from Newcastle to Morpeth. What circumstances could be more favourable, I ask, for the propagation of a contagious distemper? Yet, after most particular inquiry, I could not learn that the disease had appeared in the latter town. One gentleman, it is said, was attacked suddenly in his gig with symptoms said to indicate cholera, and died in a few hours; but this, if actually true, would be rather against the idea of contagion, since it appeared to be spontaneous in origin, and certainly was not followed by other cases.

It has been generally presumed, that cholera was conveyed from Newcastle to Haddington. But why it should have stopped exactly at this point, two stages from Edinburgh, why it did not halt at Morpeth, at Alnwick, at Belford, at Berwick-on-Tweed, &c. &c. why it did not at once proceed to Edinburgh, are points with the explanation of which we have not yet been favoured. Both in Haddington, and afterwards in Musselburgh, so far as I could learn after most diligent inquiry, the disease sprung up spontaneously, whatever be the mode in which it was afterwards propagated." 379.

Dr. Craigie adduces numerous other circumstances tending to shew that

---

* "But while we acknowledge the operation of some general epidemic cause, we cannot but observe the facility with which the disease springs up in *certain malsain localities*, and the tenacity with which it clings to *them* afterwards—thus presenting far more of the endemic than of the epidemic character."—*Med.-Chir. Rev. April* 1, 1832.

cholera is epidemic and endemic, not contagious. Its sudden invasion, and equally sudden cessation, in the places visited, are quite incompatible with the doctrine of contagion. Thus, on Wednesday the 18th of January, there was not a case of cholera in Newburn:—"on Thursday *morning* it appeared in various parts of the town and vicinity." Is this like contagion spreading from individual to individual? But why need we multiply arguments on a question which now seems to be on the point of adjudication by the general sense of mankind? With one more extract we shall close our notice of this highly interesting paper.

"As to the mode in which the disease, when once introduced, by whatever means, is diffused through a community, a few words will suffice. It must be admitted that it did run in families, two, three, or four of which were usually attacked; and this perhaps might be regarded as the strongest possible proof of contagion. This circumstance, however, must be accompanied with the qualification, that two or even three of a family were often attacked at once, or within so short a time of each other, that it is difficult to see how the disorder could have been communicated from the one to the other. I grant, that, on the period of intercourse necessary to communicate the disease, we have no exact information; and that we neither know whether one hour or one day is requisite; but we witnessed numberless instances in which the closest contact and the freest intercourse for some time was not followed by any attack. It must also be kept in view, with regard to the fact of families being attacked in Newburn, that this indicates equally the influence of the same local causes, since all were living in the same house, breathing the same air, using the same food, and exposed to the same local emanations. In one sense, indeed, the fact speaks more strongly against than in favour of contagion, since even in plague sometimes not more than one person in each family shows the marks of the pestilential poison." 379.

In conclusion, we may remark that this document from the talented Editor of the Edinburgh Medical and Surgical Journal, will do some service to the state—though it is somewhat late in its appearance. Fortunately for England, the fatal, heartless, and erroneous doctrine of contagion and importation, has done less harm, as far as human life is concerned, than in some other countries—because that horrible faith has been unable to reverse the laws of Nature, and the circumstances of society. It has done, however, incalculable mischief to commerce and domestic comfort. We will not be so uncharitable as the contagionists have been, who threatened "fire and ashes" on the heads of their opponents! No. We believe them to have been only deceived, and not designing deceivers. The very circumstance of their being so intolerant and bigotted, shews that they were *conscientious*. Do they allow so much for those who have opposed them? Alas no! From the first records of society, it has been found that, whenever the bigot took up an ERRONEOUS creed, fires, faggots, and daggers were poured on the advocates of TRUTH, with unrelenting virulence!

